# Effect of Different Mixing and Placement Methods on the Compressive Strength of Calcium-Enriched Mixture

**Published:** 2015-03-18

**Authors:** Safoora Sahebi, Nooshin Sadatshojaee, Zahra Jafari

**Affiliations:** a *Department of Endodontics, Dental School, Shiraz University of Medical Sciences, Shiraz, Iran*

**Keywords:** Calcium-Enriched Mixture, CEM cement, Compressive Strength, Mixing, Ultrasonic

## Abstract

**Introduction:** The aim of this experimental laboratory study was to evaluate the effect of different mixing and placement techniques on compressive strength (CS) of calcium-enriched mixture (CEM) cement. **Methods and Materials:** CEM powder was mixed with its liquid either by hand mixing or amalgamator mixing. The mixture was loaded to cylindrical acrylic molds with 6.0±0.1 mm height and 4.0±1 mm diameter. Half of the specimens in each group were selected randomly and ultrasonic energy was applied to them for 30 sec. All samples were incubated for 7 days at 37^°^C. The CS test was performed by means of a universal testing machine. The data were analyzed by the two-way analysis of variance (ANOVA) and Tukey’s post hoc tests. The level of significance was set at 0.05.** Results:** The maximum CS was seen in the amalgamator-mixed samples that did not receive ultrasonic agitation. The CS value of amalgamator-mixed samples was significantly higher than manually-mixed ones (*P*=0.003). Ultrasonic vibration did not change the CS of specimens. **Conclusion:** According to the results, mixing with amalgamator increases the CS of CEM cement, while ultrasonic vibration had no positive effect.

## Introduction

The mechanical and physical properties of dental materials are influenced by the mixing technique, the ratio of the constituent components, delivery system and exposure to various clinical environments [1-3]. Endodontic cements may encounter occlusal and masticatory loads, so compressive strength (CS) is amongst their important physical properties [4-6]. 

Many studies have been conducted to assess and improve physical and chemical properties of calcium silicate cements such as mineral trioxide aggregate (MTA). Basturk *et al.* [7] compared the effect of mechanical and manual mixing as well as ultrasonic agitation during placement, on CS of MTA and concluded that mechanical mixing and ultrasonic agitation enhance the CS of MTA. Shahi *et al. *[8] evaluated the effect of three different mixing methods on push-out bond strength of MTA and concluded that mixing MTA by trituration or ultrasonic energy have no significant effect on its push-out bond strength. Basturk *et al. *[9] reported that mechanical mixing of encapsulated MTA along with ultrasonic agitation has no positive effect on flexural strength and total porosity of MTA compared to manual mixing. In a study by Nekoofar *et al. *[10] they concluded that condensation pressure may affect the strength and hardness of MTA.

In 2008, calcium-enriched mixture (CEM) cement was introduced with clinical applications similar to MTA but a different chemical composition [11-14]. CEM is a tooth-colored water-based cement consisting of calcium oxide, calcium phosphate, calcium carbonate, calcium silicate, calcium sulfate, calcium hydroxide and calcium chloride [15, 16]. CEM cement exhibits favorable results regarding biocompatibility, antibacterial effect, sealing ability and setting time [15, 17-21]. 

There is little information on the effect of trituration and ultrasonic agitation on the CS of CEM cement [22]. This study was conducted to evaluate the effect of different mixing techniques and placement methods on the CS of CEM cement.

## Materials and Methods

CEM cement powder and liquid (BioniqueDent, Tehran, Iran) were mixed according to the manufacturer’s instructions. For measurement of CS, cylindrical acrylic molds with 6.0±0.1 mm height and 4.0±1 mm diameter were used and randomly allocated in four groups (*n*=10): group 1; manually-mixed and placed with ultrasonic agitation, group 2; manually-mixed and placed without ultrasonic agitation, group 3; amalgamator-mixed and placed with ultrasonic agitation and group 4; amalgamator-mixed and placed without ultrasonic agitation.

Amalgamator mixing of CEM cement was performed by mixing of 1 g CEM powder with 0.33 mL liquid in a plastic mixing capsule containing a plastic pestle at 4500 revolutions/min for 30 sec using an amalgamator (Farazmehr, Esfahan, Iran). The mixture was loaded into the molds with minimum pressure. For manual mixing, 1 g of CEM powder was mixed with 0.33 mL liquid with a spatula on a glass slab to achieve a thick creamy consistency.

Indirect ultrasonication was applied by placing an endodontic plugger (Dentsply Maillefer, Ballaigues, Switzerland) in the center of the material avoiding contact with the walls or floor of the mold and an ET20 ultrasonic tip (Satelec, Merignac, France) placed in contact with the plugger. The ultrasonic device (Suprasson P5 Booster, Satelec, France) was then activated for 30 sec at power 5. The excess material was removed.

Samples were wrapped in moistened gauze pieces and incubated at 37^°^C for 7 days. After 7 days, the specimens were removed from the molds and visually assessed for lack of air-voids and chipped edges.

The CS values were then measured by using a universal testing machine (Z020; Zwick GmbH, Ulm, Germany) at a crosshead speed of 1.0 mm/min. The maximum load needed to fracture each specimen was measured, and the CS was calculated in MPa according to the following formula: CS=4*P*/*πd*^ 2^, where *P* is the maximum load applied in Newtons (N) and *d* is the mean diameter of the specimen in mm.

The value of CS between groups was compared by using the two-way ANOVA and Tukey’s post hoc tests. The significance level was set as 0.05. The data were analyzed by SPSS software (SPSS version 18.0, SPSS, Chicago, IL, USA).

## Results

The mean±SD values for CS of CEM cement in different groups are shown in Table 1. Interaction between mixing and placement methods was not significant (*P*=0.29). Group 4 (amalgamator-mixed without ultrasonic agitation) had the maximum CS (12.52±13.44 MPa) and the minimum CS (2.10±1.14 MPa) was for group 1 (manually-mixed with ultrasonic agitation). This difference was significant (*P*=0.009). Regardless of the placement method, the mean value of CS in amalgamator-mixed groups were significantly higher than manually-mixed samples (*P*=0.003). Regardless of mixing method, method of placement had no effect on the CS of samples (*P*=0.159). In groups placed without ultrasonic agitation, amalgamator-mixed samples had significantly higher CS than manually-mixed ones (*P*=0.23).

**Table 1:** The mean (SD) and min/max values of compressive strength

**Mixing/placement technique**	**Mean (SD)**	**Min **	**Max **
**Manually/Ultrasonic**	2.10 (1.14)	0.47	3.74
**Manually/Manually**	2.93 (3.94)	0.43	12.00
**Amalgamator/Ultrasonic**	6.87 (2.13)	2.59	11.20
**Amalgamator/Manually**	12.52 (13.44)	1.27	36.90

## Discussion

This experimental laboratory study, evaluated the effect of different mixing and placement methods on CS of CEM cement. The results revealed that amalgamator mixing improved the CS of CEM cement and ultrasonic agitation had no positive effect on it.

Mixing and placement methods can affect the mechanical properties of dental materials. Amalgamator mixing and encapsulation, eliminates variations in operators ability and provides a standardized mixture.

CS is an important property of hydraulic cements that affects their clinical behavior [23]. CEM cement has various clinical applications such as perforation repair and vital pulp therapy, therefor the material should have sufficient CS to resist against the functional loads and operative procedure [24-27].

According to the results of the current study, the highest CS was recorded for amalgamator-mixed samples without ultrasonic agitation. In addition, regardless of placement method, CS of amalgamator-mixed samples, were significantly higher than manually-mixed ones. Increasing the CS of hydraulic cements by encapsulation and amalgamator mixing is in agreement with the study of Basturk *et al.* [7] who found that mechanical mixing enhanced the CS of MTA. Mechanical mixing leads to uniform and adequate wetting of powder particles and facilitates hydration process and improves the mechanical properties of the cement [9, 28]. However, Shahi *et al.* [22] reported that CS of hand mixed CEM cement was higher than its amalgamator mixed paste. This difference can be due to the differences in sample size, trituration time and time of CS evaluation. Also, they did not mention the amalgamator speed. 

In the present study, samples that were mixed manually and were placed with an ultrasonic device showed the lowest CS. This is in accordance with the results reported by Aminoshariae *et al.* [29] who found that in comparison with hand condensation, placement of MTA with ultrasonic condensation resulted in more voids in material. In fact, they concluded that ultrasonic energy pushes the MTA material against the walls of the mold and leaves voids in the body of the material. 

In the current study, ultrasonic agitation did not improve the CS of CEM cement. This finding is consistent with the study by Shahi *et al.* [8] who found that ultrasonication had no significant effect on the push-out bond strength of MTA. Also, Basturk *et al.* [9] found that ultrasonic agitation had no significant advantage in terms of total porosity and flexural strength over manual mixing of MTA. 

In contrast to our results, Basturk *et al.* [7] demonstrated that ultrasonic vibration resulted in higher CS in comparison with no ultrasonication. Different effect of ultrasonic vibration on CEM cement might be due to the differences in chemical composition and particle size of the material in comparison with MTA. Also, studies can be performed to evaluate the effect of various mixing methods and placement techniques on other physical properties of CEM.

## Conclusion

Mechanical mixing with amalgamator, increased the CS of CEM cement; however ultrasonic vibration had no effect. Further studies are needed for evaluating the effect of frequency and timing of ultrasonic vibration on CS of CEM cement. 
